# Analysis of stability and bifurcations of fixed points and periodic solutions of a lumped model of neocortex with two delays

**DOI:** 10.1186/2190-8567-2-8

**Published:** 2012-04-25

**Authors:** Sid Visser, Hil GE Meijer, Michel JAM van Putten, Stephan A van Gils

**Affiliations:** 1 Department of Applied Mathematics, University of Twente, Enschede, 7500, The Netherlands; 2 MIRA Institute for Biomedical Engineering and Technical Medicine, University of Twente, Enschede, 7500, The Netherlands; 3 Department of Clinical Neurophysiology, Medisch Spectrum Twente, Enschede, 7500, The Netherlands

## Abstract

A lumped model of neural activity in neocortex is studied to identify regions of multi-stability of both steady states and periodic solutions. Presence of both steady states and periodic solutions is considered to correspond with epileptogenesis. The model, which consists of two delay differential equations with two fixed time lags is mainly studied for its dependency on varying connection strength between populations. Equilibria are identified, and using linear stability analysis, all transitions are determined under which both trivial and non-trivial fixed points lose stability. Periodic solutions arising at some of these bifurcations are numerically studied with a two-parameter bifurcation analysis.

## 1 Introduction

 Epilepsy is a neurological disease characterized by an increased risk of recurring seizures that affects about 1% of the world population. Such seizures typically manifest themselves as brief periods in which neural activity is more synchronized than a certain baseline level. In lumped models of neural activity in the brain, these seizures are, for that reason, often characterized as large-amplitude oscillations [1]. Many causes might exist for the neural network to start oscillating, e.g., a slow parameter or an external factor might cause a bifurcation [2], or a perturbation might force the system to a different attractor [3].

**Fig. 1:**
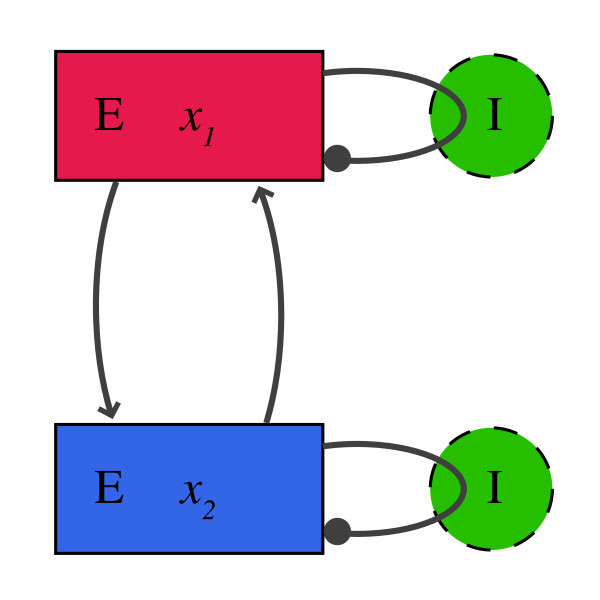
Overview of the model. Two cortical layers (*red* and *blue*) with excitatory pyramidal cells are connected mutually. The inhibition of the interneurons (*green*) is modeled intrinsically.

 In this paper, we study the attractors and their bifurcations in a lumped model of superficial and deep pyramidal cells in neocortex that has been shown to correspond well with a large detailed model whose results conformed to experiments [4,5]. The structure of this model is shown in Figure 1. Our main goal is to identify the dominating stable attractors in the system as well as their bifurcations for varying connection strength of the neural populations. The model proposed in [5] is essentially a continuous time two-node Hopfield network with discrete time delays and feedback that is governed by the following equations:

(1) $$\begin{array}{rl} \frac{dx_{1}}{dt}(t) & =-\mu_{1}x_{1}(t)-F_{1}\left(x_{1}(t-\tau_{i})\right)+G_{1}\left(x_{2}(t-\tau_{e})\right),\\ \frac{dx_{2}}{dt}(t) & =-\mu_{2}x_{2}(t)-F_{2}\left(x_{2}(t-\tau_{i})\right)+G_{2}\left(x_{1}(t-\tau_{e})\right), \end{array}$$

where $x_{i}$ is the node’s activity, $\mu_{i}$ the natural decay rate of activity, $\tau_{i}$ the time lag of feedback inhibition, $\tau_{e}$ the delay of feedforward excitation and both $F_{i}(x)$ and $G_{i}(x)$ are bounded monotonically increasing functions that represent inhibitory and excitatory synaptic activation, respectively. 

 Small Hopfield networks of this and similar forms have been studied in detail by various researches [6-22]. For example, Olien and Bélair [16] studied a two-node network with both delayed feedforward and delayed feedback connections between the nodes. Later, the same model was analyzed further by Wuan and Rei [18]. The delays in this model, however, are node-specific (the delays for all outgoing connections of a node are unique for that node) instead of connection-specific (the delays are unique for each type of connection: excitatory and inhibitory). The latter case applies to our network.

 We particularly notice the work by Shayer and Campbell [17] that studies a model very similar to the system (Equation 1) except for the fact that they choose the activation functions as odd functions. Although they numerically identify multi-stability of steady states and a periodic solution, their study mainly focuses on analytical determination of the stability and bifurcations of the trivial equilibrium in terms of the time lag parameters. In 2005, Campbell *et al.* studied the numerical continuation of periodic solutions in a ring of neurons [9]. We will extend a similar approach to a two-parameter bifurcation study in this work.

 Because Hopfield networks originate from computer science to solve mathematical programming problems [23], it is more common to study models of the Wilson-Cowan type for physiological modeling [24]. On that note, we like to point to a study by Coombes and Laing of a Wilson-Cowan type model, which is very similar to our model, in which they observe a variety of steady states, periodic solutions and chaos [25]. While Hopfield models are uncommon in mathematical neuroscience, we are not the first to study these models with a physiological relevance. For instance, Song *et al.* studied two clusters, each consisting of an excitatory and an inhibitory node that projected onto each other with delayed connections [26]. They assumed that the connections between the nodes could be faster in one direction than in the other, and they studied the model’s dependency on this difference in time lags. Furthermore, they are, to our knowledge, the only group that has performed a numerical bifurcation study of periodic orbits in two parameters for this type of model.

Due to the physiological background of our model, the delays are known and we consider fixed values of $\tau_{i}$ and $\tau_{e}$. Because of that, we are primarily interested in the parameters related to connection strength as these may be amended with anti-epileptic drugs. Although these results will depend on the chosen values of the delays, we elaborate on their robustness under variations of these delays in the discussion.

 Another difference with the pioneering works [7,17] is related to symmetry in the model. They have chosen their functions $F_{i}$ and $G_{i}$ as odd functions, which introduces a reflectional symmetry. For physiological reasons, the model considered in this paper uses non-symmetric activation functions for the synapses because the activation of synapses is thought to be stronger than the deactivation. In order to reduce the number of parameters, we choose the following:

$$\mu_{1}=\mu_{2}:=\mu ,\;F_{1}(x)=F_{2}(x):=F(x),\;G_{1}(x)=G_{2}(x):=G(x).$$

This choice of parameters and activation functions makes the model $Z_{2}$-symmetric. The following expressions are chosen for the synaptic activation functions

(2) $$F(x)=a_{i}S(\sigma_{i}x),\;G(x)=a_{e}S(\sigma_{e}x)$$

for certain *S* that is smooth, strictly increasing and satisfies S(0)=0 and $S^{\prime }(0)=1$. Typically, S(x) is bounded and sigmoidal, *i.e.S* has exactly one inflection point. The results in Section 2 are independent of the specific shape of *S*, but we will specify *S* for the numerical bifurcation analysis.

In the remaining part of this article, we study the non-dimensionalized version of Equation 1 by taking ${\tilde{x}}_{i}(\tilde{t}):=x_{i}(\mu \tilde{t})$:

(3) $$\begin{array}{rl} \frac{d{\tilde{x}}_{1}}{d\tilde{t}}(\tilde{t}) & =-{\tilde{x}}_{1}(\tilde{t})-\alpha_{1}S\left(\beta_{1}{\tilde{x}}_{1}(\tilde{t}-\tau_{1})\right)+\alpha_{2}S\left(\beta_{2}{\tilde{x}}_{2}(\tilde{t}-\tau_{2})\right),\\ \frac{d{\tilde{x}}_{2}}{d\tilde{t}}(\tilde{t}) & =-{\tilde{x}}_{2}(\tilde{t})-\alpha_{1}S\left(\beta_{1}{\tilde{x}}_{2}(\tilde{t}-\tau_{1})\right)+\alpha_{2}S\left(\beta_{2}{\tilde{x}}_{1}(\tilde{t}-\tau_{2})\right), \end{array}$$

with $\alpha_{1}:=\frac{a_{i}}{\mu }$, $\beta_{1}:=\sigma_{i}$, $\alpha_{2}:=\frac{a_{e}}{\mu }$, $\beta_{2}:=\sigma_{e}$, $\tau_{1}:=\mu \tau_{i}$ and $\tau_{2}:=\mu \tau_{e}$. For convenience, we drop the tildes from now on and switch to vector notation:

(4) $$\dot{x}(t)=f(x_{t}),\;\text{with }x_{t}\in C\left([-h,0],R^{2}\right)\text{ and }h=\max(\tau_{1},\tau_{2}).$$

In the following section, we will study this system analytically by determining its fixed points and the linear stability of these points. We will identify a stability region in parameter space and classify the bifurcations on the edge of this region. For Hopf bifurcations of the trivial steady state, we compute the first Lyapunov coefficient to study the criticality of these bifurcations. In the ‘Numerical bifurcation analysis’ section, we use software packages to determine (numerically) how the presence and stability of the bifurcating periodic solutions depend on the parameters $\alpha_{1}$ and $\alpha_{2}$.

## 2 Equilibria: linear stability and bifurcations

In this section, we study the equilibria as well as their linear stability. Necessary conditions for saddle-node, trans-critical and Hopf bifurcations are derived. Thereafter, the first Lyapunov coefficient is evaluated for the Hopf bifurcations to determine their criticality.

### 2.1 Equilibria and stability region

First we note, since S(0)=0, that the origin $\left(x_{1},x_{2})=(0,0\right)$ is always a fixed point of the system (Equation 4). For the non-trivial fixed points, the following holds:

**Theorem 1***The system* (*Equation *4) *admits exclusively symmetric fixed points*:

$$f\left(x^{*}\right)=0\;⟹\;x^{*}=\left(x^{*},x^{*}\right)\;\text{for some }x^{*}\in R.$$

*Proof* First we note that, since S(x) is a continuous strictly increasing function, its inverse function $S^{-1}(x)$ exists, and it is also continuous and strictly increasing. Next define:

$$H(x):=\frac{1}{\beta_{2}}S^{-1}\left(\frac{1}{\alpha_{2}}\left(x+\alpha_{1}S(\beta_{1}x)\right)\right).$$

Because of monotonicity of both *S* and $S^{-1}$ and positiveness of all parameters, *H* is continuous and strictly increasing as well.

Fixed points of Equation 4 satisfy $f(x^{*})=0$ which is equivalent to:

(5) $$\left\{ \begin{array}{c} x_{2}^{*}=H\left(x_{1}^{*}\right),\\ x_{1}^{*}=H\left(x_{2}^{*}\right). \end{array}\right.$$

Assume that the equilibrium is asymmetric and that $x_{1}^{*}<x_{2}^{*}$ without loss of generality. Application of *H* on both sides of this inequality and use of the conditions in Equation 5 yield:

$$x_{2}^{*}=H\left(x_{1}^{*}\right)<H\left(x_{2}^{*}\right)=x_{1}^{*}.$$

This contradicts our assumption; hence, we conclude that $x_{1}^{*}=x_{2}^{*}=x^{*}$. □

Due to the symmetric positions of these fixed points, the linearization u(t) at these equilibria takes the following form:

(6) $$\begin{array}{rl} {\dot{u}}_{1}(t) & =-u_{1}(t)-k_{1}u_{1}(t-\tau_{1})+k_{2}u_{2}(t-\tau_{2}),\\ {\dot{u}}_{2}(t) & =-u_{2}(t)-k_{1}u_{2}(t-\tau_{1})+k_{2}u_{1}(t-\tau_{2}), \end{array}$$

with

(7) $$k_{1}:=\alpha_{1}\beta_{1}S^{\prime }\left(\beta_{1}x^{*}\right),\;k_{2}:=\alpha_{2}\beta_{2}S^{\prime }\left(\beta_{2}x^{*}\right).$$

Both $k_{1}$ and $k_{2}$ take positive values only because $S^{\prime }$ is positive as well as the parameters $\alpha_{i}$ and $\beta_{i}$ for i=1,2.

 Next, we look for exponential solutions of the form $u(t)=e^{\lambda t}c$ with $c\in C^{2}$. For a non-trivial solution of Equation 6, it is required that Δ(λ)c=0, where Δ(λ) is the characteristic matrix:

(8) $$\Delta (\lambda )=\left[\begin{array}{cc} \lambda +1+k_{1}e^{-\lambda \tau_{1}} & -k_{2}e^{-\lambda \tau_{2}}\\ -k_{2}e^{-\lambda \tau_{2}} & \lambda +1+k_{1}e^{-\lambda \tau_{1}} \end{array}\right].$$

Non-trivial solutions **c** exist if the characteristic equation is satisfied:

(9) $$\begin{array}{rl} 0 & =\det\Delta (\lambda )\\ & =\underset{:=\Delta_{+}(\lambda )}{\underset{︸}{\left(\lambda +1+k_{1}e^{-\lambda \tau_{1}}+k_{2}e^{-\lambda \tau_{2}}\right)}}\underset{:=\Delta_{-}(\lambda )}{\underset{︸}{\left(\lambda +1+k_{1}e^{-\lambda \tau_{1}}-k_{2}e^{-\lambda \tau_{2}}\right)}}. \end{array}$$

From this decomposition, it follows that the spectrum of Equation 6 is the union of the spectra of the decoupled equations:

(10a) $${\dot{v}}_{-}(t)=-v_{-}(t)-k_{1}v_{-}(t-\tau_{1})+k_{2}v_{-}(t-\tau_{2}),$$

(10b) $${\dot{v}}_{+}(t)=-v_{+}(t)-k_{1}v_{+}(t-\tau_{1})-k_{2}v_{+}(t-\tau_{2}).$$

The spectra of linear DDEs with two delays, like Equations 10a and 10b, have been studied extensively since the 1960s (for instance, Bellman, Cooke and Hale [27,28]). The main consensus of these works is that the stability region often has a complex shape in terms of the parameters of the differential equation. The majority of the results in the remainder of this section and the next one (*i.e.* ‘Bifurcations’ section) could be considered as ‘common knowledge’. For the purpose of clarity, however, we have chosen to present a short derivation of these results.

We start by denoting the following theorem regarding symmetry of solutions:

**Theorem 2***Roots of*$\Delta_{-}$*correspond to symmetric solutions*, *whereas roots of*$\Delta_{+}$*relate to asymmetric solutions*.

*Proof* Let $Z_{2}$ act on $R^{2}$ so that $-1\in Z_{2}$ acts as ξ(x,y):(x,y)↦(y,x), then:

(11) $$\begin{array}{rl} \Delta_{-}(\lambda ) & =0\;\Leftrightarrow \;\{\begin{array}{c} \Delta (\lambda )v=0,\\ \eta v=v \end{array}\text{and}\\ \Delta_{+}(\lambda ) & =0\;\Leftrightarrow \;\{\begin{array}{c} \Delta (\lambda )v=0,\\ \xi v=-v. \end{array} \end{array}$$

 □

Using the characteristic equation, we can find a relation between the parameters $\left(k_{1},k_{2}\right)$ and the eigenvalues:

**Theorem 3***Let*λ=ρ+iω*for*ρ,ω∈R*satisfy the characteristic equation* (*Equation *9) *and let*$\tau_{2}>\tau_{1}>0$, *then the following inequality holds*:

(12) $$|k_{1}|+|k_{2}|\geq e^{\rho \tau }\sqrt{{\left(1+\rho \right)}^{2}+\omega^{2}},\;\tau =\{\begin{array}{cc} \tau_{2}, & \rho <0,\\ \tau_{1}, & \rho \geq 0. \end{array}$$

*Proof* Solutions of the characteristic equation (Equation 9) satisfy either $\Delta_{+}(\lambda )=0$ or $\Delta_{-}(\lambda )=0$. Upon assuming $\Delta_{+}(\lambda )=0$, it follows that:

$$e^{\max(-\rho \tau_{1},-\rho \tau_{2})}\left(|k_{1}|+|k_{2}|\right)\geq |k_{1}|e^{-\rho \tau_{1}}+|k_{2}|e^{-\rho \tau_{2}}\geq |1+\rho +i\omega |,$$

which yields the inequality (Equation 12). A similar argument for $\Delta_{-}(\lambda )$ yields the same inequality. □

**Corollary 4***An equilibrium of the system* (*Equation *4) *is asymptotically stable if*$|k_{1}|+|k_{2}|<1$.

*Proof* The inequality (Equation12) yields in this case:

(13) $$e^{\rho \tau }\sqrt{{\left(1+\rho \right)}^{2}+\omega^{2}}<1,$$

which can only hold for ρ<0. Therefore, all roots of the characteristic matrix have a negative real part and the equilibrium is asymptotically stable. □

Having obtained a minimal stability region in the Corollary 4, we study conditions for bifurcations of equilibria to expand the minimal stability region determined by Corollary 4.

### 2.2 Bifurcations

The stability of an equilibrium of a DDE is lost when one or more eigenvalues pass through the origin or the imaginary axis. The first case, in which a real eigenvalue crosses through the origin, is characterized in the following theorem:

**Theorem 5***The linearized system* (*Equation *6) *has at least one zero eigenvalue if and only if*$1+k_{1}+k_{2}=0$*or*$1+k_{1}-k_{2}=0$.

*Proof* Substitution of λ=0 into the characteristic equation (Equation 9) yields that either $\Delta_{+}(0)=0$ or $\Delta_{-}(0)=0$ and hence:

(14a) $$\Delta_{+}(0)=0\;⟹\;1+k_{1}+k_{2}=0,$$

(14b) $$\Delta_{-}(0)=0\;⟹\;1+k_{1}-k_{2}=0.$$

 □

Since the origin is always a fixed point of the system, the conditions in Theorem 5 correspond to transcritical bifurcations. For non-trivial fixed points, these conditions imply either a fold bifurcation or a trans-critical bifurcation. Because $k_{1}$ and $k_{2}$ are both positive, saddle-node bifurcations from $\Delta_{+}$ cannot occur. This, in combination with Theorem 2, leads to the conclusion that no symmetry-breaking steady-state bifurcations exist, a result which we also obtained in Theorem 1.

The case in which a pair of complex eigenvalues passes the imaginary axis is summarized in the following theorem:

**Theorem 6***Two piecewise continuous functions*$h_{+}(\omega )$*and*$h_{-}(\omega )$*exist in parameter space*$\left(k_{1},k_{2}\right)$*for which the characteristic equation* (*Equation *9) *has a pair of purely imaginary roots*λ=±iω. *Furthermore*, *when*$\omega =-\tan\omega \tau_{1}=-\tan\omega \tau_{2}$, *a line*$k_{1}+\sigma k_{2}=c$*exists for somecand*σ=±1*for which Equation *9*has roots*±iω.

*Proof* Substituting λ=iω with ω>0 into Equation 9 yields that either $\Delta_{+}(i\omega )=0$ or $\Delta_{-}(i\omega )=0$. The roots of $\Delta_{+}(i\omega )$ are considered first:

$$i\omega +1+k_{1}e^{-i\omega \tau_{1}}+k_{2}e^{-i\omega \tau_{2}}=0.$$

Splitting this equation in its real and imaginary part gives:

(15) $$\left[\begin{array}{cc} \cos(\omega \tau_{1}) & \cos(\omega \tau_{2})\\ \sin(\omega \tau_{1}) & \sin(\omega \tau_{2}) \end{array}\right]\left[\begin{array}{c} k_{1}\\ k_{2} \end{array}\right]=\left[\begin{array}{c} -1\\ \omega \end{array}\right].$$

In the case that this matrix is invertible, we find the unique solution $\left(k_{1},k_{2}\right)$ in terms of *ω* by matrix inversion:

(16) $$\left[\begin{array}{c} k_{1}\\ k_{2} \end{array}\right]=h_{+}(\omega ):=\frac{-1}{\sin(\omega (\tau_{2}-\tau_{1}))}\left[\begin{array}{cc} \sin(\omega \tau_{2}) & \cos(\omega \tau_{2})\\ -\sin(\omega \tau_{1}) & -\cos(\omega \tau_{1}) \end{array}\right]\left[\begin{array}{c} 1\\ \omega \end{array}\right].$$

In the other case, the matrix is not invertible and, hence, its determinant is zero, yielding:

(17) $$\tan\omega \tau_{1}=\tan\omega \tau_{2}.$$

Combined with the condition that ${\left[-1,\;\omega \right]}^{T}\in R(A)$, *A* being the matrix in Equation 15, follows that:

(18a) $$\omega =-\tan(\omega \tau_{1})=-\tan(\omega \tau_{2}).$$

This yields the line of solutions:

(18b) $$k_{1}+\sigma k_{2}=-\frac{1}{\cos(\omega \tau_{1})}$$

for σ=±1 such that $\cos\omega \tau_{1}=\sigma \cos\omega \tau_{2}$.

The roots of $\Delta_{-}(i\omega )$ are identified in a similar manner, yielding:

(19) $$\left[\begin{array}{c} k_{1}\\ k_{2} \end{array}\right]=h_{-}(\omega ):=\frac{-1}{\sin(\omega (\tau_{2}-\tau_{1}))}\left[\begin{array}{cc} \sin(\omega \tau_{2}) & \cos(\omega \tau_{2})\\ \sin(\omega \tau_{1}) & \cos(\omega \tau_{1}) \end{array}\right]\left[\begin{array}{c} 1\\ \omega \end{array}\right].$$

Furthermore, the same line of solutions and corresponding condition as in Equations 18a and 18b hold for $\Delta_{-}(i\omega )$. For a Hopf bifurcation to occur, any of the equations (Equations 16 to 19) must be satisfied. □

In Theorem 2, we have already shown that Hopf bifurcations caused by $\Delta_{-}$ correspond to symmetric periodic solutions. For Hopf bifurcations induced by $\Delta_{+}$, the following holds:

**Theorem 7***Hopf bifurcations corresponding with*$\Delta_{+}$*yield asymmetric periodic solutions*, *i*.*e*., $x_{1}(t)=x_{2}(t+\frac{1}{2}T)$*withTthe period of the solution*.

*Proof* Let $\lambda =i\omega_{0}$ for $\omega_{0}>0$ be a simple root of $\Delta_{+}$ (*i.e.*, of algebraic multiplicity one) and *p* a corresponding eigenvector of $\Delta (i\omega_{0})$. Then, from Hopf bifurcation theory, we know that, for *ϵ*, sufficiently small $C^{1}$ functions $k^{*}(\epsilon )$, $\omega^{*}(\epsilon )$ and $x^{*}(\epsilon )$ exist, taking values in $R^{2}$, R and $C([-h,0],R^{2})$, respectively. Furthermore, $k^{*}(\epsilon )\to h_{+}(\omega_{0})$, $\omega^{*}(\epsilon )\to \omega_{0}$ and $x(\epsilon )(t)=\epsilon ℜ(e^{i\omega^{*}(\epsilon )t}p)+o(\epsilon )$ for ϵ↓0. For $k=k^{*}(\epsilon )$ and *ϵ*, sufficiently small $\frac{2\pi }{\omega^{*}(\epsilon )}$-periodic solutions $x(t)=x^{*}(\epsilon )(t+\theta )$ exist with $\theta \in [0,2\pi /\omega^{*}(\epsilon ))$.

Since $\Delta_{+}(i\omega_{0})=0$, it follows from Equation 11 that ξp=−p. As the full non-linear equation commutes with *ξ*, it follows that the bifurcating periodic solution inherits this symmetric property:

(20) $$x\left(t+\frac{\pi }{\omega^{*}(\epsilon )}\right)=\epsilon ℜ\left(e^{i\omega^{*}(\epsilon )t+i\pi }p\right)=\xi x(t).$$

So, the condition for asymmetric periodic solutions is satisfied. □

**Fig. 2:**
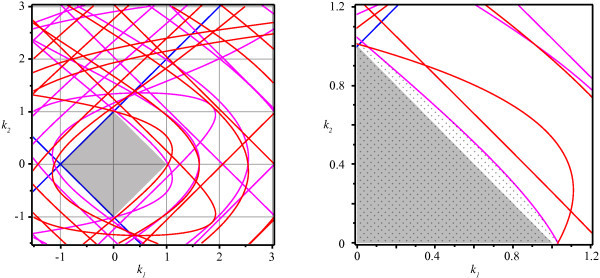
Bifurcation curves in the $\left(k_{1},k_{2}\right)$-plane. $\tau_{1}=11.6$ and $\tau_{2}=20.3$. The *right plot* shows a detail of the first quadrant only. *Blue* shows the conditions for fold or transcritical bifurcations (Equations 14a and 14b) and *red* and *magenta* depict Hopf bifurcations; equations $h_{+}$ and $h_{-}$, respectively. The *gray area* represents the stability region as in Corollary 4. The full stability region is hatched in the *right* diagram.

The different conditions for eigenvalues to have zero real part, as determined in Theorems 5 and 6, are displayed in the $\left(k_{1},k_{2}\right)$-plane in Figure 2. Due to the sine terms in the denominators of $h_{+}$ and $h_{-}$, these functions consist of numerous branches separated by asymptotes. Intersections of these curves correspond to parameters at which the system satisfies conditions for two co-dimension one bifurcations and so we expect (at least) the following co-dimension two bifurcations: Bogdanov-Takens, fold-Hopf and Hopf-Hopf. 

Studying the right diagram of Figure 2, we observe that the bifurcation curves do not coincide with the bounds of the stability region from Corollory 4. Hence, it appears that parameters exist outside this square stability region for which it still holds that all eigenvalues have negative real part. We now determine the full stability region around the origin of the $\left(k_{1},k_{2}\right)$-plane by showing that instabilities are exclusively induced by low frequencies. More precisely:

**Theorem 8***The square*$|k_{1}|+|k_{2}|<\sqrt{1+\omega_{0}^{2}}$*contains no eigenvalues*λ=±iω*for*$\omega \geq \omega_{0}>0$.

*Proof* This follows from substitution of $\lambda =i\omega_{0}$ into Theorem 3 and the fact that $\sqrt{1+\omega_{0}^{2}}$ is a monotically increasing function. □

So, if we choose $\omega_{0}$ sufficiently large as dictated by Theorem 8, no other bifurcations are located inside the bifurcation diagrams of Figure 2 for $\omega >\omega_{0}$. Hence, we can extend the stability region from the square region to the nearest bifurcation. This new stability region is hatched in the right diagram of Figure 2.

 Since we are mainly interested in stable solutions, we consider only bifurcation curves that bound the stability region. Even though we identified a bounded stability region in parameter space, we cannot assure that this is the only region in which fixed points are stable. As shown in [29], the roots of either $\Delta_{-}$ or $\Delta_{+}$ can contain multiple, disjoint regions in parameter space in which all roots have negative real parts. Since in our case, however, the eigenvalues of Equation 6 are the union of the eigenvalues of the Equations 10a and 10b, we conjecture that no other stable regions exist in parameter space than the one shown in Figure 2.

**Fig. 3:**
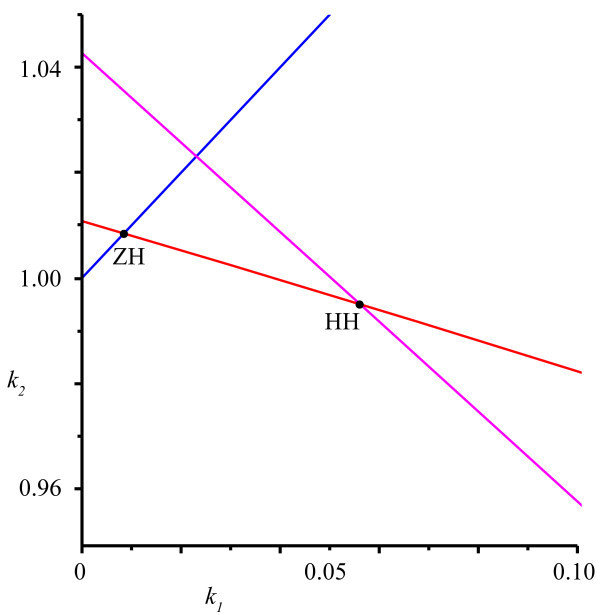
Detail of bifurcations. Similar to Figure 2 but now showing the fine structure of branches bounding the stability region. The points *ZH* and *HH* correspond with the fold-Hopf and Hopf-Hopf bifurcations from Equations 22 and 23. For clarity, we do not show the stability region. *Blue*, fold/transcritical; *red*, asymmetric Hopf; *magenta*, symmetric Hopf.

For the fixed parameters $\tau_{1}=11.6$ and $\tau_{2}=20.3$, we find that the stability region in the first quadrant is bounded by a line of fold bifurcations (Equation 14b) as well as both curves $h_{+}$ and $h_{-}$ of Hopf bifurcations; see also Figure 3. For clarity, we denote the domains of *ω* for which these curves bound the stability region by $\Omega_{S}(h_{+})$ and $\Omega_{S}(h_{-})$, respectively. We compute approximations of these ranges:

(21) $$\Omega_{S}(h_{+})\approx (0.148,0.150),\;\Omega_{S}(h_{-})\approx (0.250,0.294).$$

Similarly, we identify the codim-2 bifurcations that bound the stability region. The fold-Hopf bifurcation is located at:

(22) $$k_{\mathrm{ZH}}:=h_{+}(0.148)=\left[\begin{array}{c} 0.008\\ 1.008 \end{array}\right].$$

For the Hopf-Hopf bifurcation, we find:

(23) $$k_{\mathrm{HH}}:=h_{+}(0.150)=h_{-}(0.294)=\left[\begin{array}{c} 0.056\\ 0.995 \end{array}\right].$$

It follows from Equation 7 that, for the trivial equilibrium, the bifurcation diagram in the $\left(k_{1},k_{2}\right)$-plane determines the bifurcation diagram in the $\left(\alpha_{1},\alpha_{2}\right)$-plane up to linear rescaling.

### 2.3 The first Lyapunov coefficient

Hopf bifurcations give rise to either stable or unstable periodic solutions depending on the criticality. Therefore, we determine the first Lyapunov coefficient. Since it is easier to relate $k_{1}$ and $k_{2}$ to $\alpha_{1}$ and $\alpha_{2}$ in the origin than at non-trivial fixed points, we only consider Hopf bifurcations at the origin.

 We follow the method described in [30]. Let *p* and *q* be eigenvectors of the characteristic matrix Δ(iω) and $\Delta^{*}(i\omega )$, respectively. We normalize these vectors such that $q^{T}\Delta^{\prime }(i\omega )p=1$. By choosing $p={\left[1,1\right]}^{T}$ as an eigenvector of Δ(iω)*q* takes the form:

(24) $$q=q_{0}\left[\begin{array}{c} 1\\ 1 \end{array}\right]:=\frac{1}{2(1-k_{1}\tau_{1}e^{-i\omega \tau_{1}}+k_{2}\tau_{2}e^{-i\omega \tau_{2}})}\left[\begin{array}{c} 1\\ 1 \end{array}\right].$$

For $\phi (t)=pe^{i\omega t}$, the first Lyapunov coefficient of a (candidate) Hopf bifurcation is defined as the real part of $c_{1}$:

(25) $$\begin{array}{rcl} c_{1} & = & \frac{1}{2}q^{T}D^{3}f(0)(\phi ,\phi ,\bar{\phi })\\ & & +q^{T}D^{2}f(0)\left(e^{0\cdot }\Delta {\left(0\right)}^{-1}D^{2}f(0)(\phi ,\bar{\phi }),\phi \right)\\ & & +\frac{1}{2}q^{T}D^{2}f(0)\left(e^{2i\omega \cdot }\Delta {\left(2i\omega \right)}^{-1}D^{2}f(0)(\phi ,\phi ),\bar{\phi }\right). \end{array}$$

We note that **f** is symmetric, *i.e.*$f_{j}([x,x])=f(x)$ for j=1,2, and that it does not contain any cross terms, that is $\frac{\partial^{2}}{\partial x_{1}\;\partial x_{2}}f([x_{1},x_{2}])=0$. Therefore, both components of the differential operators $D^{2}f([x,x])$ and $D^{3}f([x,x])$ will be identical when evaluated for symmetric arguments and we denote these components by $f^{\prime \prime }(x)$ and $f^{\prime \prime \prime }(x)$, respectively. By using the multi-linear properties of the operators, we expand $c_{1}$:

(26) $$\begin{array}{rcl} c_{1} & = & \frac{1}{2}q_{0}\left[\begin{array}{cc} 1 & 1 \end{array}\right]f^{\prime \prime \prime }(0)\left(e^{i\omega t},e^{i\omega t},e^{-i\omega t}\right)\left[\begin{array}{c} 1\\ 1 \end{array}\right]\\ & & +q_{0}\left[\begin{array}{cc} 1 & 1 \end{array}\right]f^{\prime \prime }(0)\left(e^{i\omega t},e^{-i\omega t}\right)f^{\prime \prime }(0)\left(e^{0t},e^{i\omega t}\right)\Delta {\left(0\right)}^{-1}\left[\begin{array}{c} 1\\ 1 \end{array}\right]\\ & & +\frac{1}{2}q_{0}\left[\begin{array}{cc} 1 & 1 \end{array}\right]f^{\prime \prime }(0)\left(e^{i\omega t},e^{i\omega t}\right)f^{\prime \prime }(0)\left(e^{2i\omega t},e^{-i\omega t}\right)\Delta {\left(2i\omega \right)}^{-1}\left[\begin{array}{c} 1\\ 1 \end{array}\right]. \end{array}$$

Evaluation of the differential operators and the matrix inversions yields:

(27) $$\begin{array}{rcl} c_{1} & = & q_{0}(-S^{\prime \prime \prime }(0)\left(\alpha_{1}\beta_{1}^{3}e^{-i\omega \tau_{1}}-\alpha_{2}\beta_{2}^{3}e^{-i\omega \tau_{2}}\right)\\ & & +\frac{2S^{\prime \prime }{\left(0\right)}^{2}(\alpha_{1}\beta_{1}^{2}-\alpha_{2}\beta_{2}^{2})(\alpha_{1}\beta_{1}^{2}e^{-i\omega \tau_{1}}-\alpha_{2}\beta_{2}^{2}e^{-i\omega \tau_{2}})}{1+\alpha_{1}\beta_{1}-\alpha_{2}\beta_{2}}\\ & & +\frac{S^{\prime \prime }{\left(0\right)}^{2}(\alpha_{1}\beta_{1}^{2}e^{-2i\omega \tau_{1}}-\alpha_{2}\beta_{2}^{2}e^{-2i\omega \tau_{2}})(\alpha_{1}\beta_{1}^{2}e^{-i\omega \tau_{1}}-\alpha_{2}\beta_{2}^{2}e^{-i\omega \tau_{2}})}{1+2i\omega +\alpha_{1}\beta_{1}e^{-2i\omega \tau_{1}}-\alpha_{2}\beta_{2}e^{-2i\omega \tau_{2}}}). \end{array}$$

As the real part of this expression is too intricate to study analytically, we study the first Lyapunov coefficient only numerically.

 In Figure 2, we observe that, for chosen parameter $\tau_{1}=11.6$ and $\tau_{2}=20.3$, the stability region is primarily bounded by the curve $h_{-}(\omega )$ and so we study the Lyapunov coefficient along this boundary. Similarly as in [5], we choose $\beta_{1}=2$$\beta_{2}=1.2$ and

(28) $$S(x;a)=\left(\tanh(x-a)+\tanh(a)\right)\cosh^{2}(a),$$

with a=1. Values of $\alpha_{1}$ and $\alpha_{2}$ are parameterized along the boundary using Equation 7 and $\left(k_{1},k_{2}\right)$ given by $h_{-}(\omega )$ with $\omega \in \Omega_{S}(h_{-})$. In this case, we find that the first Lyapunov coefficient has a root at:

(29) $$k_{\mathrm{GH}}:=h_{-}(0.281)=\left[\begin{array}{c} 0.491\\ 0.614 \end{array}\right].$$

Such a root corresponds with a generalized Hopf bifurcation at which the criticality of the Hopf bifurcation changes. Hence, for ω<0.281, the Hopf bifurcations are supercritical and for ω>0.281 the bifurcations are subcritical.

So far, we have studied the fixed points and their bifurcations extensively, and we have shown that the system can exhibit stable periodic solutions. Since the further development of these periodic solutions cannot be studied with a local analysis of points, we must use a different approach to continue this study. Therefore, we explore the behavior of the periodic solutions numerically in the next section.

## 3 Numerical bifurcation analysis

 Here, we investigate the outcome of the periodic solutions that emanate from the Hopf bifurcations in the above text. We turn to a numerical analysis since the orbits cannot be determined analytically. More specifically, we use dde-biftool[31] to study non-trivial fixed points, and for continuation of periodic solutions, we use Knut [32]. In the following analysis, we only describe branches of solutions that are by some means associated with stable solutions. Branches not resulting in stable solutions are not discussed further.

### 3.1 One parameter bifurcations in $\alpha_{2}$

First, a bifurcation analysis is done in a single parameter. Here, we have chosen to vary the parameter $\alpha_{2}$ that represents the total amount of excitation in the system. The inhibition $\alpha_{1}$ is fixed at 0.069, and the function *S* is chosen as Equation 28 with a=1.

**Fig. 4:**
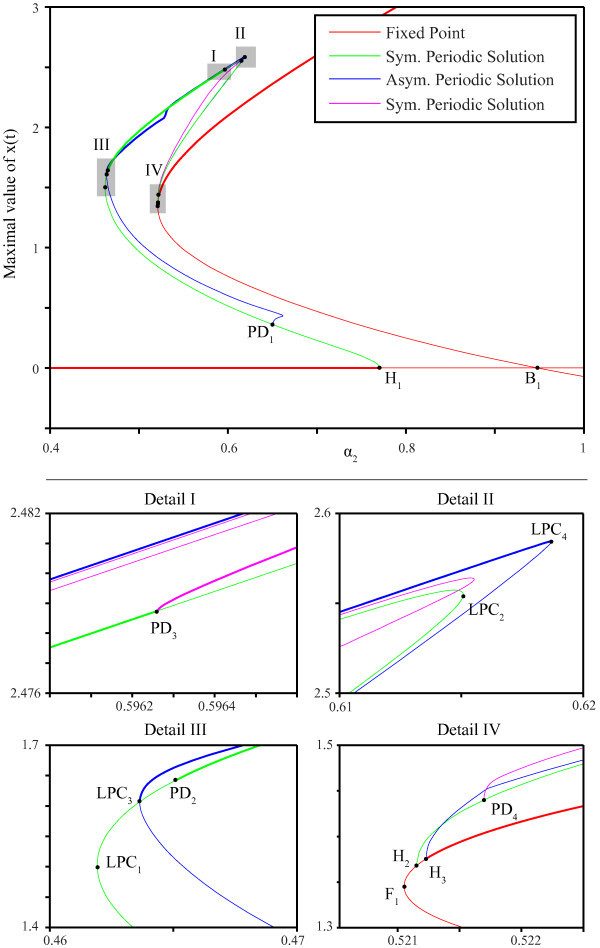
Bifurcations in one parameter. The *top* shows the bifurcation diagram in $\alpha_{2}$. Different colors represent different solutions, and a *thick*/*thinline* indicates that such a solution is stable/unstable. The four diagrams at the *bottom* show details of the four marked regions in the *top* diagram. $\tau_{1}=11.6$, $\tau_{2}=20.3$, $\alpha_{1}=0.069$, $\beta_{1}=2$, $\beta_{2}=1.2$.

The bifurcation diagram is shown in Figure 4, and corresponding parameter values for the bifurcations are shown in Table 1. Each curve represents, for different solutions, the maximum value reached during one period of the solution at different parameter values. The color corresponds with the type of solution, while thick/thin lines correspond to stable/unstable branches. 

**Table 1:** Overview of approximate parameter values for codim-1 bifurcations in $\alpha_{2}$

Point	$\alpha_{2}$
$H_{1}$	0.771
$B_{1}$	0.948
$F_{1}$	0.5211
$H_{2}$	0.5212
$H_{3}$	0.5212
$H_{4}$	1.052
${\mathrm{PD}}_{1}$	0.650
${\mathrm{LPC}}_{1}$	0.462
${\mathrm{PD}}_{2}$	0.465
${\mathrm{PD}}_{3}$	0.596
${\mathrm{LPC}}_{2}$	0.615
${\mathrm{PD}}_{4}$	0.522
${\mathrm{LPC}}_{3}$	0.464
${\mathrm{LPC}}_{4}$	0.619

Parameter $\alpha_{1}$ is fixed at 0.069.

#### 3.1.1 Fixed points

The origin is a natural starting point of our discussion of the bifurcation analysis because it is always a fixed point of Equation 4. The origin is stable until it undergoes a subcritical Hopf bifurcation $H_{1}$. Thereafter, it goes through two other Hopf bifurcations and a branch point $B_{1}$. For this value of $\alpha_{1}$, these Hopf bifurcations involve only unstable periodic solutions.

Next, we follow the fixed point that emerges from the branch point $B_{1}$. This fixed point encounters numerous Hopf bifurcations until it reaches a fold bifurcation $F_{1}$. Thereafter, it rapidly undergoes two distinct subcritical Hopf bifurcations: $H_{2}$ and $H_{3}$, becoming stable at $H_{3}$. Continuing the intersecting fixed point at $B_{1}$ in the other direction, the steady state goes through two Hopf bifurcations until it gains stability at the subcritical Hopf bifurcation $H_{4}$ (not shown).

**Fig. 5:**
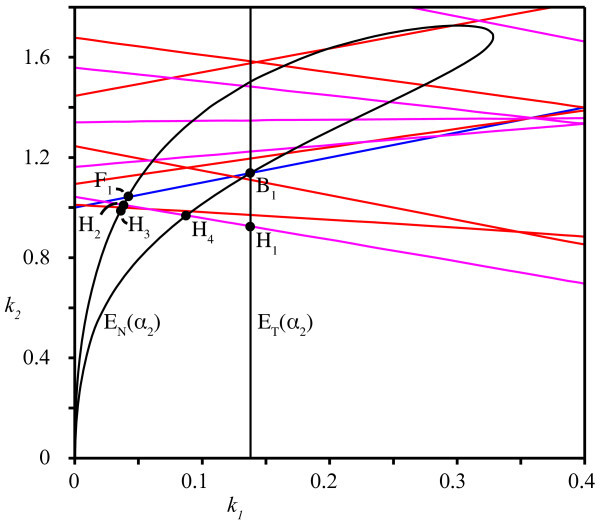
Mapping to $\left(k_{1},k_{2}\right)$-plane. The curves $E_{T}(\alpha_{2})$ and $E_{N}(\alpha_{2})$ show the parametrization of the origin (trivial fixed point) and non-trivial fixed points, respectively, for fixed $\alpha_{1}$. This figure illustrates how some solution branches can regain stability after encountering numerous bifurcations. *Blue*, fold/transcritical; *red*, asymmetric Hopf; *magenta*, symmetric periodic solution; *black*, parametrization of fixed points.

The appearance of Hopf bifurcations for fixed points is detailed in the ‘Bifucations’ section and Figure 2. If the trivial fixed point is considered, the variation of a single parameter maps the coefficients $k_{1}$ and $k_{2}$ into a straight line (Equation 7). This line, labeled $E_{T}$, is shown in the $\left(k_{1},k_{2}\right)$-plane in Figure 5. The coefficients $k_{1}$ and $k_{2}$ belonging to non-trivial equilibria, however, vary in a more complex manner when a single parameter is adjusted. Once plotted, it becomes clear that this branch encounters 18 Hopf bifurcations between departure from and return to the stability region for this specific value of $\alpha_{1}$ (see the curve $E_{N}$ in Figure 5). 

Whether a Hopf bifurcation is caused by a crossing of $\Delta_{-}$ or $\Delta_{+}$ determines whether this Hopf bifurcation results in symmetric or asymmetric periodic solutions. Hence, we conclude that Hopf bifurcations $H_{1}$, $H_{2}$ and $H_{4}$ yield symmetric periodic solutions, and that $H_{3}$ yields asymmetric ones.

#### 3.1.2 Periodic solutions

Next, we investigate the periodic solutions emanating from the Hopf bifurcations $H_{1}$, $H_{2}$ and $H_{3}$. The branch of unstable periodic solutions that emerges from $H_{1}$ consists of symmetric solutions. This matches with the analytical results since $H_{1}$ lies on $h_{-}$ and it, therefore, corresponds with symmetric solutions. The branch subsequently goes through a subcritical Neimark-Sacker bifurcation (not shown), a supercritical period-doubling bifurcation ${\mathrm{PD}}_{1}$, a limit point of cycles ${\mathrm{LPC}}_{1}$ and a subcritical period-doubling bifurcation ${\mathrm{PD}}_{2}$ at which it finally becomes stable. Then, the solution remains stable until it undergoes a supercritical period doubling bifurcation ${\mathrm{PD}}_{3}$, folds over in ${\mathrm{LPC}}_{2}$, goes through a subcritical period doubling bifurcation ${\mathrm{PD}}_{4}$ and terminates in the Hopf bifurcation $H_{2}$.

Solutions branching from ${\mathrm{PD}}_{1}$ are asymmetric. This branch folds over near ${\mathrm{PD}}_{1}$ and a second time at ${\mathrm{LPC}}_{3}$ where it gains stability. Following this branch, stability is lost at ${\mathrm{LPC}}_{4}$ and it ends in Hopf bifurcation $H_{3}$. We mention a branch sprouting from ${\mathrm{PD}}_{3}$ of symmetric solutions that is initially stable but then folds over three times before it terminates in ${\mathrm{PD}}_{4}$. Even though these solutions are initially stable, we have been unable to find these solutions in simulations because their domain of attraction is relatively small.

#### 3.1.3 Summary

**Fig. 6:**
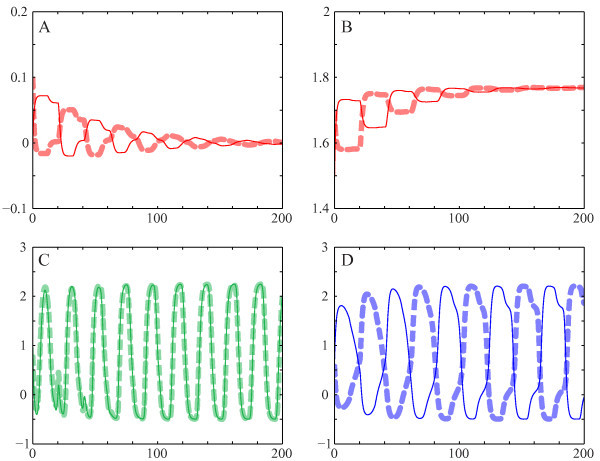
Time series in multi-stable regime. Time series of the system for $\alpha_{2}=0.55$, other parameters as in Figure 4 and initial conditions given by Equations 30a, 30b, 30c and 30d. *Solid* and *dashed lines* correspond with $x_{1}$ and $x_{2}$. Solutions of all four stable branches are obtained: **(A)** trivial steady state, **(B)** non-trivial steady state, **(C)** symmetric periodic solutions and **(D)** asymmetric periodic solutions. Colors of these time series correspond with the branches in Figure 4.

For fixed $\alpha_{1}$, we find that system can have one or two stable steady states. More specifically, for values of $\alpha_{2}$ between $H_{3}$ and $H_{1}$, two stable equilibria coexist. Stable symmetric periodic solutions exist for $\alpha_{2}$ between ${\mathrm{PD}}_{2}$ and ${\mathrm{PD}}_{3}$, and stable asymmetric periodic solutions between ${\mathrm{LPC}}_{3}$ and ${\mathrm{LPC}}_{4}$. Multi-stability of two equilibria and two periodic solutions exists for $\alpha_{2}$ between $H_{3}$ and ${\mathrm{PD}}_{3}$. This is illustrated in Figure 6 where we calculated time series of the model with fixed parameters ($\alpha_{2}=0.55$) but varying initial conditions:

(30a) $$[x_{1},x_{2}](t)=[0,0.1],$$

(30b) $$[x_{1},x_{2}](t)=[1.5,1.7],$$

(30c) $$[x_{1},x_{2}](t)=\left[1+1.2\sin\left(\frac{2\pi }{15}t\right),0.8+1.3\sin\left(\frac{2\pi }{15}t\right)\right],$$

(30d) $$[x_{1},x_{2}](t)=\left[0.7+0.7\sin\left(\frac{\pi }{30}t\right),0.6-0.9\sin\left(\frac{\pi }{30}t\right)\right],$$

with −20.3≤t≤0. All four types of limiting behavior, as determined by the preceding bifurcation analysis, are observed. 

### 3.2 Two parameter bifurcations in $\alpha_{1}$ and $\alpha_{2}$

**Fig. 7:**
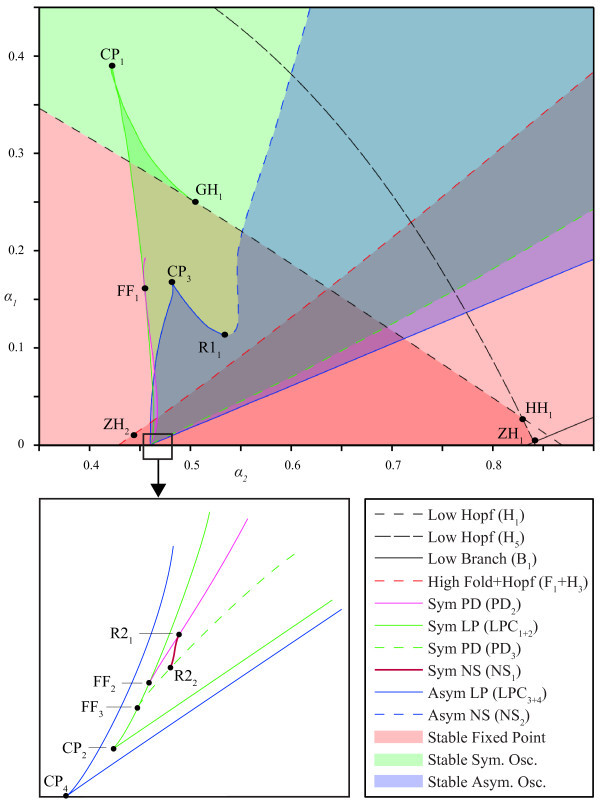
Bifurcations in two parameters. Bifurcation diagram in $\alpha_{1}$ and $\alpha_{2}$. *Colored regions* mark stability regions of indicated solutions. *Overlapping areas*, depicted with mixed colors, correspond with multi-stability. See text for a description of the points. Stability regions for individual solutions are shown in Figure 8 for clarity.

**Fig. 8:**
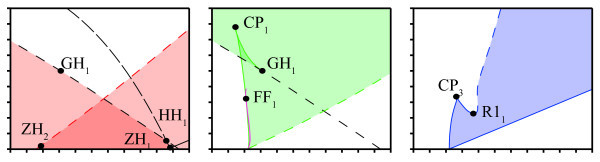
Regions of multi-stability. Identical to Figure 7, but showing the stability regions of each type of solution separately. Two partially overlapping ‘triangles’ corresponding with stability of fixed points (*left*), stability region for symmetric periodic solutions with a small area of bistability caused by cusp point ${\mathrm{CP}}_{1}$ (*middle*), and region in parameter space where stable asymmetric periodic solutions exist (*right*).

As stated before, we are mainly interested in the bifurcations at which stable solutions become unstable. These bifurcations (found with a one parameter analysis) are, therefore, continued in two parameters ($\alpha_{1}$ and $\alpha_{2}$). Figure 7 shows the relevant part of the bifurcation diagram of the system and Table 2 presents parameter values of the indicated bifurcation points. A small detail is magnified, but it shows a caricature of the complex structure. Mixed colors are used to indicate the co-existence of multiple stable solutions, but for clarity, we also show the stability regions for each type of solution separately in Figure 8. 

**Table 2:** Overview of approximate parameter values for codim-2 bifurcations in $\alpha_{1}$ and $\alpha_{2}$

Point	$\alpha_{2}$	$\alpha_{1}$
${\mathrm{HH}}_{1}$	0.829	0.028
${\mathrm{ZH}}_{1}$	0.840	0.004
${\mathrm{GH}}_{1}$	0.512	0.246
${\mathrm{ZH}}_{2}$	0.440	0.008
${\mathrm{FF}}_{1}$	0.455	0.158
*R*2_1_	0.460	2.9e-4
${\mathrm{FF}}_{2}$	0.460	2.8e-4
*R*2_2_	0.460	2.9e-4
${\mathrm{FF}}_{3}$	0.460	2.8e-4
${\mathrm{CP}}_{1}$	0.421	0.390
${\mathrm{CP}}_{2}$	0.460	2.8e-4
${\mathrm{CP}}_{3}$	0.481	0.168
*R*1_1_	0.531	0.114
${\mathrm{CP}}_{4}$	0.460	0

#### 3.2.1 Steady states

 In the one-parameter analysis, we have found that the origin and the non-trivial steady state turn unstable at Hopf bifurcations $H_{1}$ and $H_{3}$, respectively. Continuing $H_{1}$ in two parameters yields a Hopf bifurcation curve, and on this curve, we find a Hopf-Hopf bifurcation ${\mathrm{HH}}_{1}$. Following the second Hopf branch ($H_{5}$) involved, we find a transcritical-Hopf point ${\mathrm{ZH}}_{1}$ as it collides with $B_{1}$. This corresponds with the analysis of ‘Bifurcations’ section where we showed the existence of zero-Hopf and Hopf-Hopf points (see Equations 22 and 23). The arrangement of these curves is the same as in Figure 3 except for scaling. Since all involved Hopf curves at the points ${\mathrm{HH}}_{1}$ and ${\mathrm{ZH}}_{1}$ are subcritical, it follows then from the normal form analysis [33] that, for these parameters, no extra stable solutions exist near these points.

 Our analysis of the first Lyapunov coefficient also revealed the existence of a generalized Hopf bifurcation (see Equation 29). We numerically identify this point ${\mathrm{GH}}_{1}$ along the branch of $H_{1}$ by finding an emanating branch of limit point of cycles ${\mathrm{LPC}}_{1}$ with Knut. When the Hopf bifurcation $H_{3}$ of the non-trivial equilibrium is followed, a zero-Hopf bifurcation ${\mathrm{ZH}}_{2}$ is found as $H_{3}$ collides with fold bifurcation $F_{1}$. We remark that the curves $H_{3}$ and $F_{1}$ are undistinguishable in the diagram since they are close to each other for all $\left(\alpha_{1},\alpha_{2}\right)$ considered. The bifurcation ${\mathrm{ZH}}_{2}$ is a simple case ([33], s=1θ>0), yielding no additional stable solutions. These curves and the corresponding stability regions are shown in Figures 7 and 8. Bi-stability is indicated by the overlapping, darker region.

#### 3.2.2 Symmetric periodic solutions

The stability region of the symmetric periodic solutions is bounded by ${\mathrm{PD}}_{2}$ and ${\mathrm{PD}}_{3}$. Continuation of ${\mathrm{PD}}_{2}$ for stronger inhibition reveals a fold-flip bifurcation ${\mathrm{FF}}_{1}$ where the period doubling bifurcation hits ${\mathrm{LPC}}_{1}$ branch. Thereafter, it bends away and terminates. Continuing the ${\mathrm{LPC}}_{1}$ curve in the same direction, we first find a cusp point ${\mathrm{CP}}_{1}$ after which the curve ends in the generalized Hopf bifurcation ${\mathrm{GH}}_{1}$. When ${\mathrm{PD}}_{2}$ is continued in the other direction (less inhibition), it undergoes a 1:2-resonance bifurcation *R*2_1_ (*i.e.*, the period doubling branch encounters a period-doubling), and thereafter, it is subjected to a fold-flip bifurcation ${\mathrm{FF}}_{2}$ with ${\mathrm{LPC}}_{1}$. Following the ${\mathrm{LPC}}_{1}$ curve at ${\mathrm{FF}}_{2}$, we encounter another fold-flip bifurcation ${\mathrm{FF}}_{3}$ and a cusp bifurcation ${\mathrm{CP}}_{2}$. At this cusp point, the branch merges with ${\mathrm{LPC}}_{2}$.

The branch ${\mathrm{PD}}_{3}$ does not undergo any bifurcation when continued for stronger inhibition. Continuation in the other direction reveals a 1:2-resonance bifurcation *R*2_2_ and the previously identified fold-flip bifurcation ${\mathrm{FF}}_{3}$. Unfolding the 1:2-resonance bifurcations *R*2_1_ and *R*2_2_ reveals that both points are connected by the curve ${\mathrm{NS}}_{1}$ of Neimark-Sacker bifurcations. Therefore, this curve is also part of the boundary of the stability region of symmetric periodic solutions. From the unfolding of these 1:2-resonance bifurcations, we know that branches of stable homoclinic orbits should exist. However, we have been unable to continue these branches.

#### 3.2.3 Asymmetric periodic solutions

From single parameter continuation, it follows that stable asymmetric oscillations are bounded by ${\mathrm{LPC}}_{3}$ and ${\mathrm{LPC}}_{4}$. Continuation of ${\mathrm{LPC}}_{3}$ yields a cusp point ${\mathrm{CP}}_{3}$ and a 1:1-resonance bifurcation *R*1_1_ at which the branch becomes unstable. Hereafter, the stability region is bounded by a branch of Neimark-Sacker bifurcation that sprouts from *R*1_1_. When ${\mathrm{LPC}}_{3}$ is continued in the other direction, it undergoes a cusp bifurcation (${\mathrm{CP}}_{4}$) at $\alpha_{1}=0$ where ${\mathrm{LPC}}_{3}$ merges with ${\mathrm{LPC}}_{4}$.

#### 3.2.4 Summary

With the two-parameter bifurcation analysis, we find that a large part of parameter space corresponds with multi-stability. In the center, we find a region with four different stable solutions: two steady states and two periodic solutions. Furthermore, it can be seen that steady states destabilize for strong values of inhibitory feedback ($\alpha_{1}$ large) since only periodic solutions exist in the upper part of the bifurcation diagram.

### 3.3 Comparison with a realistic model

**Fig. 9:**
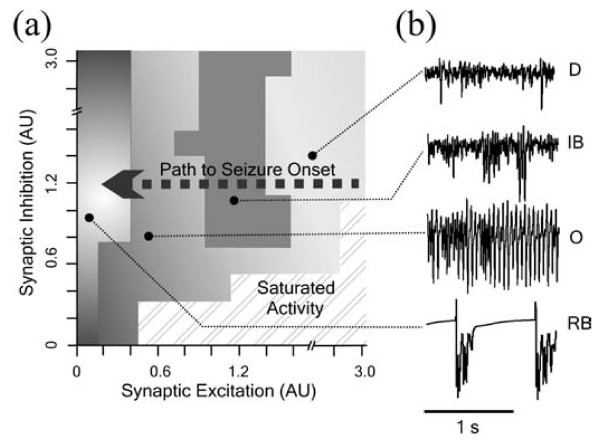
Behavior of a detailed network. This figure, copied with permission from [4], shows the behavioral changes of a large physiologically detailed model of neocortex. For varying strengths of excitatory and inhibitory connections, the model’s behavior is classified in one of five categories. See text for a description of the network states and their correspondence to the population model.

 As this two-parameter bifurcation study might seem contrived for a fairly simple model, we like to make a comparison with a study of a more biologically realistic model. Van Drongelen *et al.* analyzed a small model of neocortex consisting of 656 neurons to study emergent epileptiform activity [4]. For similar reasons as in this study, they varied only parameters related to excitatory and inhibitory synaptic strength, and they then obtained Figure 9. In this figure, the behavior of their realistic model for different choices of parameters is categorized in one of five categories: desynchronized, irregular bursting, oscillatory, regular bursting and saturated activity. The small exemplary time series show for each class the characteristic behavior of the model except for saturated activity. This latter state is best described as a state in which all neurons are non-stop activated in an incoherent manner. 

 In the regular bursting state, the model is rather quiet apart from a burst of activity that occurs regularly about every second. These bursts are primarily generated by slow dynamical processes in the underlying neurons. In the absence of slow processes, the network would exhibit no activity in this state [4]. Hence, this type of behavior should be compared with the trivial steady state of our model. Furthermore, the non-trivial steady state in our simplified model corresponds with saturated activity in the detailed model because the network is very active, but no clear oscillations or rhythms are observed. Finally, the oscillatory state can be compatible with both the symmetric and asymmetric periodic solutions in our model.

With these analogues for the observed types of network behavior in our mind, the bifurcation diagram in Figure 7 displays several strong similarities with the detailed network model in Figure 9. For low excitation, both models exhibit regular bursting/trivial steady-state solutions. Furthermore, we see in both cases a triangular region at the bottom in which both models exhibit saturated/non-trivial steady-state solutions. Finally, we observe that the above-mentioned regions are separated by a regime of oscillatory solutions. We also note that not all types of behavior in the detailed model have a counterpart in the simplified model, but we will elaborate on this in the discussion.

## 4 Discussion

 In this paper, we have studied a continuous time two-node Hopfield network with two discrete time delays. The model has been derived in [5], and it describes the activity of two excitatory neural populations located in different layers of the mammalian neocortex. Inhibitory connections are assumed to exist only between neurons within the same population, whereas excitatory connections are exclusively made between both populations. Furthermore, a bifurcation study in the same article has shown that the model is able to produce different types of behavior that correspond to a realistic 656-neuron model of neocortex as proposed in [34]. This detailed model is able to reproduce phenomena observed in *in vitro* experiments in mouse [4]. By studying the population model more thoroughly, we hope to gain a better understanding of the complex dynamics seen in the realistic 656-neuron model. In this way, new experiments for both *in silico* and *in vitro* environments can be proposed.

Even though Hopfield networks of this and similar forms have been studied thoroughly in other works, these works mainly consider changes of the dynamics under variation of the time delays. As the time lags in our model are fixed because of the physiological background, we are mainly interested in the dynamics’ dependency on connectivity parameters. As a new contribution to this field, we have focused our study of the model on varying connection strengths of excitatory and inhibitory connections.

All the bifurcations that we have identified in the model, both analytically and numerically, satisfied the non-degeneracy condition. Combined with the fact that the model depends smoothly on all parameters, all these bifurcations are structural. Hence, local variations of parameters will result in local variations of the bifurcations and the stability region. Some of the delicate bifurcation structures that we identified will be more sensitive to parameter variations, but only because of their limited separation in parameter space.

For the steady states in the model, we have analytically determined conditions in terms of the coupling parameters for which these states become unstable due to bifurcations. We have found that both the trivial and the non-trivial equilibria undergo fold as well as Hopf bifurcations. The non-trivial equilibria, however, are the solution of a transcendental equation, and therefore, we have studied these bifurcations numerically. In this manner, we have identified a region in parameter space of bi-stability in which both the trivial and a non-trivial fixed point are stable.

By studying the first Lyapunov coefficient at the Hopf bifurcations in the system, we have found both supercritical and subcritical bifurcations. Furthermore, we have analytically determined the type of bifurcating periodic solution, either symmetric (in-phase) or asymmetric (anti-phase) oscillations. The evolution of the periodic solutions arising at the Hopf bifurcations is studied numerically with continuation software. A large region in parameter space is determined in which both types of periodic solutions co-exist. Furthermore, we have identified numerous codim-2 bifurcations: cusp, generalized Hopf, zero-Hopf, Hopf-Hopf, fold-flip and both 1:1 and 1:2 resonance bifurcations. In the area where bistability exists between these different solutions, simulations have shown that the solutions often tend to the asymmetric solutions.

 Combining the stability regions of the steady states and the periodic solutions, we have found a region in parameter space in which four types of stable solutions co-exist: the trivial fixed point, a non-trivial fixed point and both symmetric and asymmetric periodic solutions. Although it has been shown in [35] that small Hopfield networks can exhibit chaotic behavior, we have not found such behavior in this study.

 The biological relevance of these results is, in our opinion, significant as well. We have shown that the complex bifurcation structure of the model matches with the dynamical changes seen in a biologically relevant model for variations of both excitatory and inhibitory strengths [4]. This relation is most clear for the regular bursting, oscillatory and saturated states of the detailed model because these have a clear equivalent attractor in the population model studied in this article. The other states of the detailed network, however, might be produced by the population model as part of a transient behavior. Long transients, during which the model resides close to several attractors for an extended period of time, are not uncommon for multi-stable delayed systems. Since these transients are not attractors themselves, they cannot be identified with a bifurcation analysis as in this study. Therefore, it remains to be determined whether the population model exhibits long transients and, if so, whether the time-series correspond with the two missing network states, *i.e.*, desynchronized and irregular bursting as described in [4].

Although the considered model has very little resemblance with the structures of a real brain, we still believe that studying models like these provide new insights. Complex bifurcation structures and multi-stability observed in these models reveal possible transitions of network behavior that might not have been considered before. For that reason, we plan to seek and analyze such critical transitions more accurately with a detailed model of neuronal activity.

Furthermore, we plan to investigate networks of similar systems in order to study emergent patterns. It is promising that the combined analytical/numerical study of a single column already shows interesting dynamics, in particular, multi-stability. We expect to find patterns in such networks that will be relevant to understand observed patterns in slice experiments.

## Competing interests

The authors declare that they have no competing interests.

## Authors’ contributions

SV and SvG conceived the study, carried out the analysis and drafted the manuscript, HM participated in the numerical bifurcation analysis, and SvG and MvP participated with coordination and interpretation. All authors read and approved the final manuscript.

## References

[B1] Lytton WW Computer modelling of epilepsy Nat Rev, Neurosci 2008 9 626 637 10.1038/nrn2416 18594562 PMC2739976

[B2] Wendling F Bartolomei F Bellanger JJ Chauvel P Epileptic fast activity can be explained by a model of impaired GABAergic dendritic inhibition Eur J Neurosci 2002 15 1499 1508 10.1046/j.1460-9568.2002.01985.x 12028360

[B3] Lopes da Silva FH Blanes W Kalitzin SN Parra J Suffczynski P Velis DN Epilepsies as dynamical diseases of brain systems: basic models of the transition between normal and epileptic activity Epilepsia 2003 44 72 83 10.1111/j.0013-9580.2003.12005.x 14641563

[B4] Van Drongelen W Lee HC Hereld M Chen Z Elsen FP Stevens RL Emergent epileptiform activity in neural networks with weak excitatory synapses IEEE Trans Neural Syst Rehabil Eng 2005 13 236 241 10.1109/TNSRE.2005.847387 16003905

[B5] Visser S Lee HC Meijer HGE van Drongelen W van Putten MJAM van Gils SA Comparing epileptiform behavior of meso-scale detailed models and populations models of neocortex Clin Neurophysiol 2010 27 471 478 10.1097/WNP.0b013e3181fe0735 21076324

[B6] Baldi P Atiya A How delays affect neural dynamics and learning IEEE Trans Neural Netw 1994 5 4 612 621 10.1109/72.298231 18267834

[B7] Bélair J Campbell SA Stability and bifurcations of equilibria in a multiple-delayed differential equation SIAM J Appl Math 1994 54 1402 1424 10.1137/S0036139993248853

[B8] Campbell SA Edwards R Van den Driessche P Delayed coupling between two neural network loops SIAM J Appl Math 2004 65 316 335 10.1137/S0036139903434833

[B9] Campbell SA Yuan Y Bungay SD Equivariant Hopf bifurcation in a ring of identical cells with delayed coupling Nonlinearity 2005 18 2827 2846 10.1088/0951-7715/18/6/022

[B10] Campbell SA Ncube I Wu J Multistability and stable asynchronous periodic oscillations in a multiple-delayed neural system Physica D, Nonlinear Phenom 2006 214 2 101 119 10.1016/j.physd.2005.12.008

[B11] Chen Y Wu J Minimal instability and unstable set of a phase-locked periodic orbit in a delayed neural network Physica D, Nonlinear Phenom 1999 134 2 185 199 10.1016/S0167-2789(99)00111-6

[B12] Faria T On a planar system modelling a neuron network with memory J Differ Equ 2000 168 129 149 10.1006/jdeq.2000.3881

[B13] Guo S Huang L Hopf bifurcating periodic orbits in a ring of neurons with delays Physica D, Nonlinear Phenom 2003 183 1-2 19 44 10.1016/S0167-2789(03)00159-3

[B14] Guo S Chen Y Wu J Two-parameter bifurcations in a network of two neurons with multiple delays J Differ Equ 2008 244 2 444 486 10.1016/j.jde.2007.09.008

[B15] Huang L Wu J Nonlinear waves in networks of neurons with delayed feedback: pattern formation and continuation SIAM J Math Anal 2003 34 4 836 860 10.1137/S0036141001386519

[B16] Olien L Bélair J Bifurcations, stability, and monotonicity properties of a delayed neural network model Physica D, Nonlinear Phenom 1997 102 3-4 349 363 10.1016/S0167-2789(96)00215-1

[B17] Shayer LP Campbell SA Stability, bifurcation, and multistability in a system of two coupled neurons with multiple time delays SIAM J Appl Math 2000 61 673 700 10.1137/S0036139998344015

[B18] Wei J Ruan S Stability and bifurcation in a neural network model with two delays Physica D, Nonlinear Phenom 1999 130 3-4 255 272 10.1016/S0167-2789(99)00009-3

[B19] Wei JJ Velarde MG Bifurcation analysis and existence of periodic solutions in a simple neural network with delays Chaos 2004 14 3 940 953 10.1063/1.1768111 15447004

[B20] Wu J Faria T Huang YS Synchronization and stable phase-locking in a network of neurons with memory Math Comput Model 1999 30 1-2 117 138 10.1016/S0895-7177(99)00120-X

[B21] Yuan Y Campbell SA Stability and synchronization of a ring of identical cells with delayed coupling J Dyn Differ Equ 2004 16 709 744 10.1007/s10884-004-6114-y

[B22] Yuan Y Dynamics in a delayed-neural network Chaos Solitons Fractals 2007 33 2 443 454 10.1016/j.chaos.2006.01.018

[B23] Wen UP Lan KM Shih HS A review of Hopfield neural networks for solving mathematical programming problems Eur J Oper Res 2009 198 3 675 687 10.1016/j.ejor.2008.11.002

[B24] Wilson HR Cowan JD Excitatory and inhibitory interactions in localized populations of model neurons Biophys J 1972 12 1 24 4332108 10.1016/S0006-3495(72)86068-5 PMC1484078

[B25] Coombes S Laing C Delays in activity-based neural networks Philos Trans R Soc Lond Ser A, Math Phys Sci 2009 367 1117 1129 10.1098/rsta.2008.0256 19218154

[B26] Song Y Makarov VA Velarde MG Stability switches, oscillatory multistability, and spatio-temporal patterns of nonlinear oscillations in recurrently delay coupled neural networks Biol Cybern 2009 101 147 167 10.1007/s00422-009-0326-5 19629517

[B27] Bellman R Cooke KL Differential-Difference Equations 1963 Academic Press, San Diego

[B28] Hale JK Functional Differential Equations 1971 Springer, Berlin

[B29] Mahaffy JM Zak PJ Joiner KM A geometric analysis of stability regions for a linear differential equation with two delays Int J Bifurc Chaos Appl Sci Eng 1995 5 3 779 10.1142/S0218127495000570

[B30] Diekmann O van Gils SA Verduyn-Lunel SM Walther HO Delay Equations: Functional-, Complex-, and Nonlinear Analysis 1995 Springer, New York

[B31] Engelborghs K Luzyanina T Roose D Numerical bifurcation analysis of delay differential equations using DDE-BIFTOOL ACM Trans Math Softw 2002 28 1 21 10.1145/513001.513002

[B32] Roose D Szalai R Continuation and bifurcation analysis of delay differential equations Numerical Continuation Methods for Dynamical Systems: Path Following and Boundary Value Problems 2007 Springer, Berlin 359 399

[B33] Kuznetsov YA Elements of Applied Bifurcation Theory 2004 Springer, New York

[B34] Van Drongelen W Koch H Elsen FP Lee HC Mrejeru A Doren E Marcuccilli CJ Hereld M Stevens RL Ramirez JM Role of persistent sodium current in bursting activity of mouse neocortical networks in vitro J Neurophysiol 2006 96 2564 2577 10.1152/jn.00446.2006 16870839

[B35] Lu H Chaotic attractors in delayed neural networks Phys Lett A 2002 298 2-3 109 116 10.1016/S0375-9601(02)00538-8

